# TRIBE-2: a phase III, randomized, open-label, strategy trial in unresectable metastatic colorectal cancer patients by the GONO group

**DOI:** 10.1186/s12885-017-3360-z

**Published:** 2017-06-09

**Authors:** Chiara Cremolini, Federica Marmorino, Fotios Loupakis, Gianluca Masi, Carlotta Antoniotti, Lisa Salvatore, Marta Schirripa, Luca Boni, Vittorina Zagonel, Sara Lonardi, Giuseppe Aprile, Emiliano Tamburini, Vincenzo Ricci, Monica Ronzoni, Filippo Pietrantonio, Chiara Valsuani, Gianluca Tomasello, Alessandro Passardi, Giacomo Allegrini, Samantha Di Donato, Daniele Santini, Alfredo Falcone, on behalf of all the investigators of the Gruppo Oncologico del Nord Ovest

**Affiliations:** 1 Dipartimento di Ricerca Traslazionale e delle Nuove Tecnologie in Medicina e Chirurgia, Oncologia Medica 2 Universitaria, Azienda Ospedaliero-Universitaria Pisana, Via Roma 67, 56126 Pisa, Italy; 2 Centro per il Coordinamento per le Sperimentazioni Cliniche, Istituto Toscano Tumori, AOU Careggi, viale Pieraccini 6, 50139 Florence, Italy; 3 Oncologia Medica 1, Istituto Oncologico Veneto, Istituto di Ricovero e Cura a Carattere Scientifico (IRCCS), via Gattamelata 64, 35128 Padua, Italy; 4 Dipartimento di Oncologia, Azienda Ospedaliero-Universitaria Santa Maria della Misericordia, Piazzale Santa Maria della Misericordia 15, 33100 Udine, Italy; 5 Ospedale Infermi, Via Coriano 38, 47924 Rimini, Italy; 6 Oncologia Medica, Dipartimento di Oncologia Clinica, Azienda Sanitaria Ospedaliera S. Croce, Via Michele Coppino 26, 12100 Cuneo, Italy; 7 Ospedale San Raffaele, Via Olgettina 60, 20132 Milan, Italy; 8 Fondazione I.R.C.C.S, Istituto Nazionale dei Tumori, Via Venezian 1, 20133 Milan, Italy; 9 Dipartimento Oncologico, A.S.L. 1 Carrara, P.zza Sacco e Vanzetti, 2, 54033 Carrara, Italy; 10 SC Oncologia, Istituti Ospedalieri Di Cremona, Viale Concordia 1, 26100 Cremona, Italy; 11 Dipartimento di Oncologia Medica, IRCCS-IRST (Istituto Scientifico Romagnolo per lo Studio e la Cura dei Tumori), via Piero Maroncelli 40, 47014 Meldola, FC Italy; 12 Oncologia Medica, Ospedale Felice Lotti, via Roma 147, 56025 Pontedera, Italy; 13 Dipartimento Oncologico, AUSL 4 Prato, Via Mazzamuti 7, 59100 Prato, Italy; 14 Oncologia Medica, Università Campus Bio-Medico, vai Alvaro del Portillo 200, 00128 Rome, Italy

**Keywords:** Colorectal cancer, Bevacizumab, Strategy, Folfoxiri, Clinical trial

## Abstract

**Background:**

Chemotherapy plus bevacizumab is a standard first-line treatment for unresectable metastatic colorectal cancer patients. Different chemotherapy backbones may be chosen, including one to three drugs, based on patients’ general conditions and comorbidities, treatments’ objectives, and disease characteristics. TRIBE trial demonstrated a significant advantage in terms of progression-free survival and overall survival for FOLFOXIRI plus bevacizumab as compared with FOLFIRI plus bevacizumab. Based on recent evidence, the de-intensification of the upfront regimen after 4–6 months of treatment is nowadays regarded as a valuable option. Moreover, the prolonged inhibition of angiogenesis, and in particular the continuation of bevacizumab beyond the evidence of disease progression, is an efficacious strategy in the treatment of metastatic colorectal cancer patients.

**Methods/design:**

TRIBE-2 is a prospective, open-label, multicentric phase III randomized trial in which unresectable and previously untreated metastatic colorectal cancer patients are randomized to receive first-line FOLFOX plus bevacizumab followed by FOLFIRI plus bevacizumab after disease progression or FOLFOXIRI plus bevacizumab followed by the re-introduction of the same regimen after disease progression. The primary endpoint is to compare the efficacy of the two proposed treatment strategies in terms of Progression Free Survival 2.

**Discussion:**

The TRIBE-2 study aims at answering the question whether the upfront use of FOLFOXIRI improves the clinical outcome of metastatic colorectal cancer patients, when compared with the pre-planned, sequential use of oxaliplatin-based and irinotecan-based doublets. Both proposed treatment strategies are designed to exploit the effectiveness of the prolonged inhibition of angiogenesis, alternating short (up to 4 months) induction periods and less intensive maintenance phases.

**Trial registration:**

TRIBE2 is registered at Clinicaltrials.gov: NCT02339116. January 12, 2015. TRIBE-2 is registered at EUDRACT 2014–004436-19, October 10, 2014.

**Electronic supplementary material:**

The online version of this article (doi:10.1186/s12885-017-3360-z) contains supplementary material, which is available to authorized users.

## Background

### FOLFOXIRI plus bevacizumab as first-line treatment of unresectable mCRC

The first-line treatment is a crucial starting point in the therapeutic route of every metastatic colorectal cancer (mCRC) patients [1]. The strategic value of this choice lies in the importance of obtaining disease control, preventing disease progression and achieving symptoms’ relief. Moreover, the first-line treatment allows pursuing the unique chance of cure for a percentage of patients, and exploiting subsequent interventions, in terms of both surgical/locoregional approaches and other systemic treatments. In the last few years, many therapeutic associations emerged as possible options and the selection of the most appropriate treatment is a challenging issue for medical oncologists.

Recent evidences demonstrate that the intensity of chemotherapy can be modulated: traditional oxaliplatin or irinotecan-based doublets [2, 3] can be de-potentiated in fluoropyrimidine monotherapy [4, 5] or intensified in the triple regimen FOLFOXIRI (5-FU, oxaliplatin and irinotecan) [6, 7].

In particular, the phase III randomized TRIBE study [6] strengthened the use of FOLFOXIRI plus bevacizumab (bev) as a new option for the upfront treatment of mCRC patients who meet inclusion criteria of the study. This multicenter trial included 508 patients with unresectable mCRC to compare FOLFOXIRI plus bev with FOLFIRI plus bev. Patients received up to 12 cycles of induction treatment, followed by maintenance therapy with 5-FU/LV (5-fluorouracil/leucovorin) and bev until disease progression. The trial met its primary endpoint progression free survival (PFS). Indeed, the triplet plus bev provided a significant advantage in terms of PFS as compared with FOLFIRI (folinic-acid, 5-Fluorouracil, irinotecan) plus bev (median PFS: 12.1 months versus 9.7 months, HR 0.75, 95% CI 0.62–0.90; *P* = 0.003). After an extended median follow-up period of 48.1 months, updated results demonstrated a significant advantage also in terms of overall survival (OS) (median OS: 29.8 months versus 25.8 months; HR 0.80, 95% CI 0.65–0.98; *P* = 0.030). Subgroup analyses evidenced no significant interaction between treatment effect and baseline clinical characteristics or *RAS* and *BRAF* molecular status [8].

Second and further lines of treatment were chosen by investigators. The 76% of patients in both arms were exposed to second-line treatments and around 80% of patients in the experimental arm received again a fluoropyrimidine +/− oxaliplatin +/− irinotecan as part of their second-line therapy. Of note, while induction treatments were administered up to 6 months, the median PFS in the FOLFOXIRI plus bev group was longer than 12 months, so that the median duration of oxaliplatin- and irinotecan-free intervals was around 6 months.

### Maintenance therapy after chemotherapy plus bevacizumab

The optimal duration of chemotherapy and bev is still a matter of debate [9–11]. Results from recent randomized studies showed that the de-intensification of the chemotherapy backbone while continuing the antiangiogenic is an efficacious strategy. Maintenance allows to delay progression, thus prolonging the time interval between the completion of the induction treatment and the evidence of disease progression. These longer “full chemotherapy”-free intervals make more clinically and biologically sound the reintroduction of agents used during the induction phase after the occurrence of progression.

Three studies compared maintenance strategies following chemotherapy plus bev with clinical observation.

SAKK 41/06 [12] was a non-inferiority trial that randomized patients that did not progress after 4–6 months of XELOX (capecitabine/oxaliplatin) or FOLFOX (folinic-acid, 5-Fluorouracil, oxaliplatin) plus bev, to continue or not bev alone until disease progression. The non-inferiority of the observation strategy was not demonstrated in terms of both time to progression (TTP) and OS.

In the CAIRO-3 trial [13], patients achieving disease stabilization or response after six cycles of CAPOX (capecitabine/oxaliplatin) plus bev were randomized between observation and maintenance treatment with “low dose” capecitabine plus bev. After the first disease progression, CAPOX plus bev had to be reintroduced and continued until the second evidence of disease progression. The primary endpoint was PFS2, defined as the time from randomization to progression upon re-introduction of CAPOX plus bev. Patients in the maintenance arm achieved a significant benefit in terms of PFS2, PFS and a non-significant advantage in OS.

Finally, AIO KRK 0207 study [14] investigated whether treatment discontinuation or continuation with bev alone was non-inferior to maintenance with fluoropyrimidine/bev, in mCRC patients who had received 24 weeks of an oxaliplatin-based doublet. At first progression, re-induction of the initial treatment was planned. The primary endpoint was time to failure of strategy (TFS). Results from this trial showed that bev monotherapy was not inferior to a fluoropyrimidine plus bev as maintenance, while the non-inferiority was not demonstrated for the observation strategy in terms of TFS. At the time of data analysis, OS results were extremely immature.

On the basis of these evidences, the opportunity to alternate induction and maintenance phases in the disease history of mCRC patients is nowadays a valuable option.

### Continuation of bevacizumab beyond progression in mCRC

More than 10 years ago, preclinical evidence led to hypothesize that continuing antiangiogenic treatments beyond the occurrence of resistance could be efficacious in mCRC. Results from the observational studies BRiTE [15] and ARIES [16] provided initial clinical data supporting this suggestion.

The prospective confirmation of the effectiveness of the prolonged inhibition of angiogenesis was provided by the phase III ML18147 [17] and BEBYP trials [18].

ML18147 study [17], conducted in Europe and Saudi Arabia, randomIzed mCRC patients previously treated with bev plus a first-line doublet to the switched doublet with or without bev. Eligible patients were those who had experienced progressive disease up to 3 months after discontinuing first-line bev plus chemotherapy. The use of bev beyond progression provided a significant advantage in terms of OS, primary endpoint, (11.2 vs 9.8 months, HR: 0.81 [0.69–0.94], *p* = 0.0062) and PFS (5.7 vs 4.1 months, HR: 0.68 [0.59–0.78], *p* < 0.0001).

BEBYP trial [18], contemporaneously conducted in Italy and prematurely stopped when results from ML18147 were released, had a similar design. This study evaluated the reintroduction of bev even in patients who had completed the first line treatment more than 3 months before disease progression to first-line. The continuation of bev beyond progression provided significant benefit in terms of PFS, primary endpoint, (6.8 vs 5.0 months, HR: 0.72 [0.54–0.97], *p* = 0.0029), while no statistically significant differences in OS (14.1 vs 15.5 months, HR: 0.77 [0.56–1.07], *p* = 0.12) were reported. Nevertheless, the trial was clearly underpowered to detect an advantage in terms of survival. The benefit in PFS in favor of the continuation of bev was observed in all analyzed subgroups and in particular both in patients who discontinued bev for more or less than 3 months.

Based on these data, the continuation of bev beyond progression is an efficacious option in the treatment of mCRC patients.

Drawing from these evidences the phase III TRIBE-2 study was designed in order to compare two first- and second-line strategies, aiming at exploiting the effectiveness of a prolonged inhibition of angiogenesis. A standard strategy of an upfront doublet (FOLFOX) plus bev followed by a switched doublet (FOLFIRI) plus bev after disease progression is compared to a strategy of upfront FOLFOXIRI plus bev then followed by reintroduction of FOLFOXIRI plus bev after disease progression. All combination treatments are repeated up to 8 cycles and followed by maintenance with a fluoropyrimidine plus bev, in order to shorten the duration of more intensive treatments and to prolong as more as possible oxaliplatin- and irinotecan-free intervals.

## Methods/design

### Study treatment

**Fig. 1:**
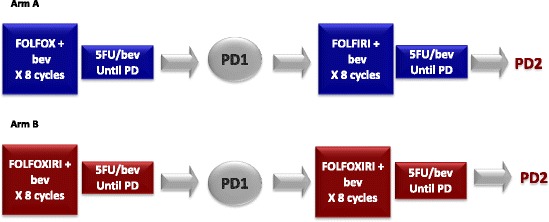
Study treatment

This is a prospective, open-label, multicentric phase III randomized trial in which initially unresectable and previously untreated mCRC patients are randomized to receive two different strategies: first-line FOLFOX plus bev followed by FOLFIRI plus bev after disease progression (arm A, standard treatment) or first-line FOLFOXIRI plus bev followed by reintroduction of FOLFOXIRI plus bev after disease progression (arm B, experimental strategy). All treatments are administrated up to 8 cycles followed by 5-FU/LV plus bev maintenance until disease progression, unacceptable adverse events, or consent withdrawal (Fig. 1).

**Fig. 2:**
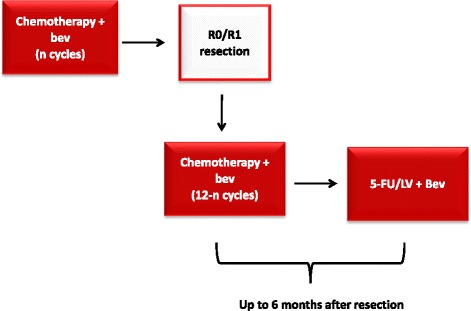
Recommended post-operative treatment in the case of radical resection of metastases

In the case of surgical radical resection of residual metastases, post-operative therapy with the same pre-operative regimen is planned up to an overall duration of 6 months (12 cycles), then followed by 5FU/LV plus bev up to 6 months after resection. This choice lies on the rationale that, though in the absence of a formal demonstration, it might be reasonable that if a regimen has allowed to pursue secondary resection in a specific patient, it will be also effective in preventing disease relapse (Fig. 2).

### Study objectives and endpoints

TRIBE-2 study aims at comparing the efficacy of the two proposed treatment strategies: the upfront use of the triplet plus bev followed by the reintroduction of the same regimen at disease progression or the sequential use of oxaliplatin-based and irinotecan-based doublets, combined with the antiangiogenic.

The primary endpoint of this trial is Progression Free Survival 2 (PFS2), beginning with randomization and ending with the first of the following events: a) death; b) disease progression on any treatment given after 1st progression.. Disease status will be evaluated according to RECIST 1.1 criteria [19]. The determination of disease progression will be based on investigator-reported measurements and a subsequent central revision is planned.

For patients that will not receive any treatment within 3 months after 1st progression, PFS2 will be equal to PFS, since in these patients the proposed treatment strategy has failed; on the other hand, if following a RECIST but clinically irrelevant disease progression, investigators decide to wait until the subsequent disease assessment and the time interval is <3 months, this is accepted as part of the strategy.

Secondary objectives of this study are to compare the two proposed treatment strategies in terms of: PFS, 2nd-PFS,time to failure of strategy, OS, response rate, rate of secondary R0 resection of metastases, safety profile.

2nd-PFS is defined as the time from the start of the second-line treatment to the evidence of disease progression or death from any cause, whichever occurs first.

TFS is defined as the time from randomization to the first of the following events: death, introduction of a new drug (a drug that was not included in the original strategy), evidence of disease progression during treatment with all the drugs of the initial strategy.

### Statistical design

Based on the assumption that PFS2 of each arm follows an exponential distribution and hypothesizing a true HR for PFS2 of 0.77 between the experimental and the control group, 466 events are required for a two-sided unstratified log-rank test to have 80% power with α = 0.05. Assuming a proportion of PFS2 equal to 50% at 15 months in arm A, this treatment effect can be translated to a 9% absolute improvement in PFS2 at 15 months in arm B. With an accrual rate of 200 subjects/year, a minimum follow up period equal to 1.5 years and an overall dropout rate of 5%, it is required to enroll 654 subjects, randomized in a 1:1 ratio.

A group sequential design with one interim analysis is planned. The analyses will take place at 2/3 (303 events) of the events for the primary endpoint, using an O’Brien Fleming alpha-spending rule. The interim analysis will assess the superiority of the experimental arm versus the control group for the primary endpoint: the study will be considered for early termination only if superiority is met. The first interim analysis will have a two-sided alpha level of 0.0131. According to the O’Brien Fleming spending rule this will leave a two-sided alpha level of 0.0455 for the final analysis. Therefore, the total type I error rate will be only slightly increased.

### Study population

As in previous trials by the GONO group (Table 1) investigating the use of the triplet, only patients between 18 and 75 years old are eligible. Those between 71 and 75 years old are included only if their ECOG performance status is 0. Main eligibility criteria include measurable disease according to RECIST version 1.1 [19], adequate bone marrow, liver, and renal function, no previous exposure to an oxaliplatin-containing adjuvant therapy. Previous adjuvant chemotherapy with fluoropyrimidines alone is allowed if more than 6 months have elapsed between the end of the adjuvant therapy and disease relapse. Other exclusion criteria include: peripheral neuropathy of grade 2 or higher according to NCI-CTCAE version 4.0 [20] and contraindications to study drugs.

**Table 1:** TRIBE-2 participating centers

Principal Investigator	Site Name	City
Sergio Bracarda	Ospedale San Donato	Arezzo
Angela Buonadonna	Centro di Riferimento Oncologico	Aviano
Nicola Silvastris	IRCCS BARI	Bari
Stefania Mosconi	A.O. Papa Giovanni XXIII	Bergamo
Alberto Zaniboni	Fondazione Poliambulanza Istituto Ospedaliero	Brescia
Saverio Cinieri	Ospedale “SENATORE A. PERRINO”	Brindisi
Andrea Mambrini	A.S.L. 1 Massa Carrara	Carrara
Roberto Bordonaro	A.O.R.N.A.S. Garibaldi	Catania
Gianluca Tomasello	Istituti Ospitalieri di Cremona	Cremona
Cristina Granetto	Azienda Sanitaria Ospedaliera Santa Croce e Carle	Cuneo
Carlo Milandri	A.U.S.L. 11 Empoli	Empoli
Antonio Frassoldati	Azienda Ospedaliero Universitaria di Ferrara	Ferrara
Francesco Di Costanzo	Azienda Ospedaliero-Universitaria Careggi	Firenze
Angela Stefania Ribecco	P.O. S. Giovanni di Dio	Firenze
Luisa Fioretto	Ospedale Santa Maria Annunziata AUSL 10 FI	Firenze
Teresa Gamucci	Polo Oncologico Provinciale Frosinone Azienda Sanitaria Locale	Frosinone
Silvana Chiara	IRCCS AOU San Martino	Genova
Alberto Sobrero	IRCCS Ospedale San Martino IST	Genova
Matteo Clavarezza	E.O. Ospedali Galliera	Genova E.O.G.
Carmelo Bengala	A.U.S.L. 9 Grosseto	Grosseto
Laura Scaltriti	Azienda USL di Reggio Emilia	Guastalla
Carlo Aschele	Ospedale Felettino ASL 5 Spezzino	La Spezia
Gianpiero Romano	Ospedale Vito Fazzi	Lecce
Antonio Ardizzoia	A.O. Provincia di Lecco	Lecco
Andrea Bonetti	A.U.L.S.S. 21 Legnago	Legnago
Samanta Cupini	Ospedali Riuniti di Livorno	Livorno
Giovanni Luca Frassineti	IRCCS-Istituto Scientifico Romagnolo per lo Studio e la Cura dei Tumori (I.R.S.T.)	Meldola
Filippo De Braud	Istituto Nazionale dei Tumori	Milano
Mauro Moroni	Ospedale San Carlo Borromeo	Milano
Luca Gianni	Ospedale San Raffaele	Milano
Chiara Carlomagno	Azienda Ospedaliera Universitaria Federico II	Napoli
Sara Lonardi	Istituto Oncologico Veneto	Padova
Livio Blasi	Ospedale “CIVICO - DI CRISTINA - BENFRATELLI”	Palermo
Francesca Pucci	Azienza Ospedaliera Universitaria	Parma
Silvia Brugnatelli	Fondazione I.R.C.C.S. Policlinico San Matteo	Pavia
Enrichetta Corgna	Azienda Ospedaliera di Perugia	Perugia
Alfredo Falcone	Azienda Ospedaliero Universitaria Pisana	Pisa
Giacomo Allegrini	Ospedale Felice Lotti Pontedera	Pontedera
Samantha Di Donato	A.U.S.L. 4 Prato	Prato
Maria Banzi	Arcispedale Santa Maria Nuova	Reggio Emilia
Emiliano Tamburini	Ospedale Infermi	Rimini
Enrico Cortesi	Policlinico Umberto I	Roma
Domenico Cristiano Corsi	Ospedale San Giovanni Calibita Fatebenefratelli Isola Tiberina	Roma
Daniele Santini	Policlinico Unversitario Campus Bio-Medico	Roma
Clementina Savastano	OORR S.Giovanni di Dio e Ruggi d’Aragona	Salerno
Marco Benasso	A.S.L. 2 Savonese	Savona
Francesco Di Clemente	A.U.S.L. 7 Siena	Siena
Alessandro Bertolini	Azienda Ospedaliera della Valtellina e della Valchiavenna	Sondrio
Patrizia Racca	Azienda Ospedaliero-Universitaria San Giovanni Battista di Torino	Torino
Massimo Aglietta	Istituto di Candiolo IRCCS	Torino
Stefania Miraglia	AslTO1	Torino
Giuseppe Aprile	A.O. Universitaria Santa Maria della Misericordia	Udine
Giampaolo Tortora	Azienda Ospedaliera Universitaria Integrata	Verona
Domenico Amoroso	Ospedale Versilia	Viareggio
Enzo Maria Ruggeri	AUSL Viterbo - Ospedale Belcolle	Viterbo

### Study procedures

Patients enrolled in arm A receive up to 8 cycles of m-FOLFOX6 plus bev, then followed by maintenance with 5FU/LV plus bev at the same dosage used in the last cycle of m-FOLFOX6 plus bev. In the case of disease progression they receive up to 8 cycles of FOLFIRI plus bev, then followed by maintenance with 5FU/LV plus bev at the same dosage used in the last cycle of FOLFIRI plus bev. Patients enrolled in arm B receive up to 8 cycles of FOLFOXIRI plus bev, then followed by maintenance with 5FU/LV plus bev at the same dosage used in the last cycle of FOLFOXIRI plus bev. In the case of disease progression during maintenance, FOLFOXIRI plus bev will be reintroduced up to 8 cycles, followed by 5FU/LV plus bev as maintenance. If disease progression occurs during FOLFOXIRI plus bev a second-line treatment at investigator’s choice will be administered. Doses of cytotoxics adopted in the second-line treatments may be modified based on adverse events occurred during first-line. If the planned treatment after progression will be deemed unfeasible, modified regimens, including doublets plus bev in the triplet arm, are allowed and patients will be evaluable for the primary endpoint.

Disease assessment is performed every 8 weeks by means of CT scan.

### Safety

The investigator is responsible for ensuring that all adverse events observed by the investigator or reported by subjects are properly captured in the subjects’ medical records and in electronic Case Report Forms (eCRFs). It is left to the investigator’s clinical judgment to determine whether an adverse event is related and of sufficient severity to require the subject’s removal from treatment or from the study.

All serious adverse events (SAEs) occurring during the study treatment period, defined as through to 6 months after the last dose of the treatment or the end of the study whichever is longer, must be reported within 24 h. The investigator should notify the Sponsor of all SAEs in accordance with local procedures, statutes and the European Clinical Trial Directive (where applicable). The Sponsor is responsible for the medical review of all SAEs and for their notification to the appropriate Ethics Committees, Competent Authorities and participating Investigators, in accordance with local requirements and the European Clinical Trial Directive.

The same procedures are applied to the following serious and non serious Adverse Events of Special Interest (AESI) to bev: hypertension ≥ grade 3; proteinuria ≥ grade 3; gastrointestinal perforation, abscesses and fistulae (any grade); wound healing complications ≥ grade 3; haemorrhage ≥ grade 3 (any grade Central Nervous System bleeding; ≥ grade 2 haemoptysis); arterial thromboembolic events (any grade); venous thromboembolic events ≥ grade 3; posterior reversible encephalopathy syndrome (any grade); congestive hearth failure ≥ grade 3; non-gastrointestinal fistula or abscess ≥ grade 2.

### Quality

Each participating Investigator is responsible for ensuring data quality as planned in the Data Validation Plan document. Periodic monitoring visits at participating centers are planned. All information are systematically checked for consistency, completeness and accuracy by the Coordinating Data Center (Clinical Trials Coordinating Center, Istituto Toscano Tumori), that periodically issues Data Query Forms in case of inconsistent data. Local quality control is provided by qualified monitors, responsible for the consistency of data reported in eCRFs.

### Translational analyses

An extensive program of translational analyses is planned. The availability of tissue specimens (primary tumor or metastatic site) is mandatory for study entry. Tissue specimens are collected for the central assessment of *RAS* and *BRAF* status and for further molecular analyses. Not only samples available at baseline, but also those obtained during the treatment (i.e. in the case of secondary resections) are centrally collected. No additional samples, other than those obtained for clinical purposes, are requested.

Given the growing relevance of techniques able to monitor the dynamism of tumor evolution across time and therapies, blood and plasma samples are collected at baseline, and at the first and second evidence of disease progression.

### Ethics

The protocol is conducted in accordance to the standards of Good Clinical Practice, in agreement with the principles laid down by the 18th World Medical Assembly (Helsinki, 1964) and subsequent amendments (Tokyo 1975, Venice, 1983, Hong Kong, 1989, Somerset West, 1996 and Edinburgh, 2000).

The study (Protocol v.2.1, 2nd September 2014) was approved in December 2014 by the Ethics Committee of the Coordinating Center (Comitato Etico Area Vasta Nord Ovest, CEAVNO) and then approved by the local Ethics Committees of participating centers. All candidate patients will provide their informed consent to study procedures before enrollment in the study.

## Discussion

Results from the TRIBE study [6] clearly underlined the role of FOLFOXIRI plus bev as upfront option for unresectable mCRC patients, confirming the superiority of the triplet over the doublet FOLFIRI when bev is added to both regimens. Based on these evidences, the triplet plus bev is now a widely accepted treatment option, supported by all major clinical guidelines [21] (http://www.nccn.org/professionals/physician_gls/pdf/colon.pdf).

The TRIBE-2 study aims at fully answering the question of whether the upfront use of FOLFOXIRI improves the clinical outcome of mCRC patients, when compared with the pre-planned, sequential use of oxaliplatin-based and irinotecan-based doublets.

In order to shorten the duration of combination treatments and to exploit the progression delaying ability of bev-based maintenance, combination regimens are restricted to short induction periods (4 months instead of 6 months previously adopted in the TRIBE study) then followed by maintenance with fluoropyrimidine plus bev until tumor progression.

Another phase II study by the GONO group, named MOMA (NCT02271464), recently evaluated the efficacy of a 4 months induction treatment with the triplet plus bev, then followed by maintenance. In this fase II trial, mCRC patients were randomized to receive up to 8 cycles of first-line FOLFOXIRI plus bev, followed by bev alone until disease progression, or the same upfront treatment, followed by bev plus metronomic capecitabine plus cyclophosphamide [22]. The accrual was completed in March 2015 and results will be available in 2016.

Though acknowledging that a formal demonstration of PFS2 surrogacy for OS is currently lacking, the choice of PFS2 as primary endpoint of the TRIBE-2 study reflects the objective to prospectively compare two strategies, with pre-planned upfront regimens and treatments after progression. As specified in the study protocol, PFS2 is defined as the time from randomization to disease progression on any treatment given after first progression, or death, whichever occurs first. While the study will provide clear evidence about the feasibility and the efficacy of the experimental strategy of reintroduction of FOLFOXIRI plus bev after progression, the rationale for considering in the definition of PFS2 “any treatment given after first progression”, and not only those included in the pre-planned treatment strategies, lies in the objective to pragmatically catch the impact of the upfront use of FOLFOXIRI plus bev, independently of the actual re-introduction of the same regimen after progression.

## Additional file

Additional file 1: List of Ethics Committes that approved the study protocol. (DOCX 116 kb)

## Abbreviations

5-FU: 5-fluorouracil
AE: Adverse Event
AESI: Adverse Events of Special Interest
Bev: Bevacizumab
CAPOX: Capecitabine/Oxaliplatin
CT: Computed Tomography
CTCAE: Common Terminology Criteria for Adverse Events
e.g.: Example given
ECOG PS: Eastern Cooperative Oncology Group – Performance Status
e-CRF: Electronic Case Report Form
EGFR: Epidermial Growth Factor Receptor
FOLFIRI: Folinic-acid, 5-Fluorouracil, irinotecan
FOLFOX: Folinic-acid, 5-Fluorouracil, oxaliplatin
FOLFOXIRI: Folinic-acid, 5-Fluorouracil, oxaliplatin, irinotecan
GI: Gastrointestinal
GONO: Gruppo Oncologico Nord-Ovest
HR: Hazard Ratio
ITT: Intention To Treat
LOHP: oxaliplatin
LV: Leucovorin
mCRC: Metastatic colorectal cancer
NA: Not Available
NCI CTCAE: National Cancer Institute Common Terminology Criteria for Adverse Events
OS: Overall survival
PD: Progression Disease
PFS: Progression Free Survival
PR: Partial Response
RECIST: Response Evaluation Criteria in Solid Tumors
RR: Response rate
SAE: Serious Adverse Event
TFS: Time to Failure of Strategy
TTP: Time To Progression
