# Supplementary material for: TRIBE-2: a phase III, randomized, open-label, strategy trial in unresectable metastatic colorectal cancer patients by the GONO group
Source: BMC Cancer. 2017 Jun 9;17:408. doi: 10.1186/s12885-017-3360-z (PMC5466800; doi:10.1186/s12885-017-3360-z)
Supplement: Additional file 1: — List of Ethics Committes that approved the study protocol. (DOCX 116 kb) [file 12885_2017_3360_MOESM1_ESM.docx]

**List of Ethics Committes that approved the study protocol**

| **Site** | **Center** | **Responsible Ethics Committe** |
| --- | --- | --- |
| Pisa | Azienda Ospedaliero Universitaria Pisana | Comitato Etico Area Vasta Nord Ovest |
| Cuneo | Azienda Sanitaria Ospedaliera Santa Croce e Carle | Comitato Etico Interaziendale ASO S. Croce e Carle e AA.SS.LL CN1. CN2, AT Via Monte Zovetto 18 12100 Cuneo |
| Torino | Azienda Ospedaliero-Universitaria San Giovanni Battista di Torino | COMITATO ETICO INTERAZIENDALE A.O.U. CITTA' DELLA SALUTE E DELLA SCIENZA DI TORINO A.O. ORDINE MAURIZIONE A.S.L. TO1 |
| Bergamo | Humanitas Gavazzeni | Comitato Etico dell'IRCCS - Istituto Clinico Humanitas - Via Manzoni 56 - Rozzano (MI) |
| Brescia | Fondazione Poliambulanza Istituto Ospedaliero | Comitato Etico Provinciale Provincia di Brescia e/o Spedali Civili |
| Cremona | Istituti Ospitalieri di Cremona | COMITATO ETICO PER LE PROVINCE DI CREMONA MANTOVA E LODI |
| Lecco | A.O. Provincia di Lecco | Comitato Etico per le  Province di Lecco Como e Sondrio |
| Milano | Istituto Nazionale dei Tumori | Comitato Etico Fondazione IRCCS  Istituto Nazionale Tumori |
| Milano | Ospedale San Carlo Borromeo | Comitato Etico  Interaziendale Milano Atrea A |
| Milano | Ospedale San Raffaele | IRCCS OSPEDALE SAN RAFFAELE |
|  |  |  |
| Monza | Azienda Ospedaliera San Gerardo | COMITATO ETICO DELLA PROVINCIA MONZA BRIANZA |
| Pavia | Fondazione I.R.C.C.S. Policlinico San Matteo | Comitato di Bioetica |
| Sondrio | Azienda Ospedaliera della Valtellina e della Valchiavenna | Comitato etico Interaziendale per le Province di Lecco, Como e Sondrio |
| Trento | Ospedale di Trento / Azienda Provinciale per i Servizi Sanitari | Segretaria del comitato Etico  per le sperimentazioni cliniche |
| Legnago | A.U.L.S.S. 21 Legnago | Comitato Etico per la Sperimentazione  clinica delle province di verona e rovigo |
| Padova | Istituto Oncologico Veneto | CESC dell'IRCCS Istituto Oncologico Veneto |
| Verona | Azienda Ospedaliera Universitaria Integrata | Comitato Etico Per la Sperimentazione clinica (CESC) delle province di verona e rovigo con sede presso AOUI di Verona |
| Aviano | Centro di Riferimento Oncologico | Comitato Etico Regionale Unico |
| Udine | A.O. Universitaria Santa Maria della Misericordia | Comitato Etico Regionale Unico |
| Bologna | Azienda Unità Sanitaria Locale di Bologna | Comitato Etico Interaziendale  Bologna-Imola CE-BI |
| Bologna | A.O. Universitaria Policlinico Sant’Orsola Malpighi | Ricerca ed Innovazione- Casadio- Comitato Etico (Pad 3) |
| Ferrara | Azienda Ospedaliero Universitaria di Ferrara | Comitato Etico della Provincia di Ferrara |
| Parma | Azienza Ospedaliera Universitaria | Comitato Etico per Parma |
| Reggio Emilia | Arcispedale Santa Maria Nuova | COMITATO ETICO PROVINCIALE |
| Rimini | Ospedale Infermi | Comitato Etico IRST-IRCCS e Area Vasta Romagna |
| Genova | E.O. Ospedali Galliera | IRCCS Azienda Ospedaliera Universitaria San Martino – IST Istituto Nazionale per la Ricerca sul Cancro |
| Genova | IRCCS AOU San Martino Chiara | IRCCS Azienda Ospedaliera Universitaria San Martino – IST Istituto Nazionale per la Ricerca sul Cancro |
| La Spezia | Ospedale Felettino ASL 5 Spezzino | Comitato Etico  Regionale Liguria |
| Savona | A.S.L. 2 Savonese | Comitato Etico Azienda  Ospedaliera Universitaria S. Martino di Genova |
| Arezzo | Ospedale San Donato | Segreteria Scientifica CEAVSE AUSL 8 Arezzo Ufficio per la ricerca clinica e profili etici/giuridici |
| Carrara | A.S.L. 1 Massa Carrara | Comitato Etico Area Vasta Nord Ovest |
| Empoli | A.U.S.L. 11 Empoli | Comitato Etico  Area Vasta Centro |
| Firenze | Azienda Ospedaliero-Universitaria Careggi | Area Vasta Centro |
| Firenze | Ospedale Santa Maria Annunziata AUSL 10 FI | Coordinamento Comitati etici aziendali |
| Grosseto | A.U.S.L. 9 Grosseto | CEAVSE-Sezione Azienda USL 9 |
| Livorno | Ospedali Riuniti di Livorno | Comitato Etico Area Vasta Nord Ovest |
| Pontedera | Ospedale Felice Lotti Pontedera | CE AVNO |
| Prato | A.U.S.L. 4 Prato | Comitato Etico Area  Vasta Centro |
| Siena | A.U.S.L. 7 Siena | Comitato Etico per la Sperimentazione Clinica  dei Medicinali Area Vasta Sud-Est |
| Viareggio | Ospedale Versilia | CEAVNO |
| Perugia | Azienda Ospedaliera di Perugia | SEAS Umbria |
| Roma | Policlinico Umberto I | dei Medicinali Area Vasta |
| Roma | Ospedale San Giovanni Calibita Fatebenefratelli Isola Tiberina | Comitato Etico Lazio 1 |
| Roma | Policlinico Unversitario Campus Bio-Medico | Comitato Etico dell'Università Campus Bio Medico |
| Latina | Ospedale Santa Maria Goretti | Comitato Etico Lazio 2 |
| Frosinone | Polo Oncologico Provinciale Frosinone Azienda Sanitaria Locale | Comitato Etico Lazio 2 |
| Napoli | Azienda Ospedaliera Seconda Università Degli Studi di Napoli (SUN) | Comitato Etico Seconda  Università degli Studi di Napoli |
| Napoli | Azienda Ospedaliera Universitaria Federico II | Comitato Etico Federico II |
| San Giovanni Rotondo | I.R.C.C.S. Casa Sollievo della Sofferenza | Segreteria Comitato Etico Direzione Sanitaria IRCCS Casa Sollievo della Sofferenza Viale Cappuccini 1 71013 San Giovanni Rotondo (FG) |
| Lecce | Ospedale Vito Fazzi | Comitato Etico |
| Caltanissetta | P.O. Sant'Elia | Comitato Etico Palermo 1 |
| Catania | A.O.R.N.A.S. Garibaldi | Comitato Etico Catania 2 |
| Forlì | IRCCS  Istituto Scientifico Romagnolo per lo Studio e la Cura dei Tumori (I.R.S.T.) | COMITATO ETICO IRST IRCCS e AVR |
| Reggio Emilia | Azienda USL di Reggio Emilia Ospedale di Guastalla | COMITATO ETICO PROVINCIALE DI REGGIO EMILIA |
| Firenze | P.O. S. Giovanni di Dio | Coordinamento Comitati etici aziendali |
| Bari | IRCCS BARI | Comitato Etico Istituto  Oncologico "Giovanni Paolo II" |
| Bergamo | A.O. Papa Giovanni XXIII | Comitato Etico della Provincia di Bergamo |
| Palermo | OSPEDALE "CIVICO - DI CRISTINA - BENFRATELLI" | Comitato Etico Palermo 2 |
| Salerno | OORR S.Giovanni di Dio e Ruggi d'Aragona | Comitato Etico Campania Sud |
| Torino | Istituto di Candiolo IRCCS | Comitato Etico IRCCS Candiolo |
| Torino | AslTO1 | Inviare tutto alla data manager |
| Genova | IRCCS AOU San Martino Sobrero | IRCCS Azienda Ospedaliera Universitaria San Martino – IST Istituto Nazionale per la Ricerca sul Cancro |
| Roma | Policlinico Universitario  Tor Vergata | Comitato Etico Indipendente Fondazione PTV-Policlinico Tor Vergata |
| Chieti | P.O. SS. Annunziata | Comitato Etico delle Province di Chieti e Pescara e dell'Università degli studi "G. D'Annunzio" di Chieti-Pescara |
| Viterbo | AUSL Viterbo - Ospedale Belcolle | Comitato Etico Lazio 1 |
| Brindisi | P.O. "Senatore A. Perrino" | Comitato Indipendente di Etica Medica ASL BR |
| Sesto San Giovanni | Ist. Clinici di perfezionamento | Comitato Etico Milano Area C |
| Milano | Azienda Ospedaliera San Paolo | Comitato Etico Interaziendale  Milano Area A |
| Genova | Ballestrero | Comitato Etico Regione Liguria _Sezione 2 IRCCS Azienda Ospedalia Universitaria San Martino-IST Largo Rosanna Benzi- 16132 |
